# Expression in Sf9 insect cells, purification and functional reconstitution of the human proton-coupled folate transporter (PCFT, SLC46A1)

**DOI:** 10.1371/journal.pone.0177572

**Published:** 2017-05-11

**Authors:** Swapneeta S. Date, Mariana C. Fiori, Guillermo A. Altenberg, Michaela Jansen

**Affiliations:** 1 Department of Cell Physiology and Molecular Biophysics, School of Medicine, Texas Tech University Health Sciences Center, Lubbock, TX, United States of America; 2 Center for Membrane Protein Research, School of Medicine, Texas Tech University Health Sciences Center, Lubbock, TX, United States of America; University of Bern, SWITZERLAND

## Abstract

The proton-coupled folate transporter (PCFT) provides an essential uptake route for the vitamin folic acid (B9) in mammals. In addition, it is currently of high interest for targeting chemotherapeutic agents to tumors due to the increased folic acid requirement of rapidly dividing tumor cells as well as the upregulated PCFT expression in several tumors. To understand its function, determination of its atomic structure and molecular mechanism of transport are essential goals that require large amounts of functional PCFT. Here, we present a high-level heterologous expression system for human PCFT using a recombinant baculovirus and *Spodoptera frugiperda* (Sf9) insect cells. We demonstrate folate transport functionality along the PCFT expression, isolation, and purification process. Importantly, purified PCFT transports folic acid after reconstitution. We thus succeeded in overcoming heterologous expression as a major bottleneck of PCFT research. The availability of an overexpression system for human PCFT provides the basis for future biochemical, biophysical and structural studies.

## Introduction

Folate, pteroyl-L-glutamate, also known as vitamin B9, plays crucial roles in more than a hundred metabolic reactions in mammalian cells. Folates are required for DNA and amino acid synthesis, DNA repair, synthesis of the 1-carbon donor S-adenosyl methionine, and other methylation reactions [1]. An adequate supply of folates is thus essential for normal development and proliferation of cells. Bacteria and other unicellular organisms can synthesize folic acid *de novo* [2], but most eukaryotes, including humans, lack the enzyme dihydropteroate synthase, which is required for *de novo* folate synthesis [3, 4]. Human cells are thus dependent on transport mechanisms to provide folates for metabolic needs. Absorption of folates through the diet is the only natural source of folates in mammals. This absorption is mediated in the duodenum and upper jejunum by the proton-coupled folate transporter (PCFT) that functions optimally at the prevalent physiologic pH of 5–5.5 [5]. PCFT belongs to the major facilitator superfamily (MFS) of transporters. As is characteristic of MFS transporters, PCFT contains a 12 transmembrane helical arrangement with both N- and C-termini oriented intracellularly [6–8]. Loss-of-function mutations in the gene encoding PCFT, SLC46A1, manifest in the form of hereditary folate malabsorption, clearly demonstrating that PCFT represents the molecular entity responsible for intestinal folate uptake [9–17]. Consistent with its role in DNA and protein synthesis, higher quantities of folates are required in rapidly diving cells such as during pregnancy and in cancers [18, 19], and substantial levels of PCFT have been found in tumor cells of breast, prostate and ovarian cancers, providing an entry pathway for antifolate anticancer agents into cells [20–25].

One of the primary requirements for detailed structural and functional studies of proteins is the availability of sufficiently high yields for biochemical and biophysical studies; large quantities of purified membrane proteins are required for high-resolution structural studies such as X-ray crystallography. In the case of PCFT, mammalian cell lines and *Xenopus laevis* oocytes have been established as expression systems [9, 26, 27]. However, limitations in scale-up and/or insufficient protein yields reduce the utility of these expression systems. Here, we report the expression and purification of human PCFT using the baculovirus/*Spodoptera frugiperda* (Sf9) insect cells system to produce functional PCFT in sufficient quantities for detailed biochemical, biophysical and structural studies.

## Experimental procedures

### Reagents

TALON Cobalt Resin was purchased from Clontech Laboratories, Inc. (Mountain View, CA). The antibody against the His tag (THE^TM^ Anti-His mAb) was purchased from GenScript (Piscataway, NJ). The tritiated folic acid derivative (folic acid, diammonium salt, [3',5',7,9-^3^H], 19.4 Ci/mmol) was purchased from Moravek Biochemicals Inc. (Brea, CA). For gel chromatography Mini-PROTEAN TGX (Tris-Glycine eXtended) Precast gels (BioRad, Hercules, CA) were used, the stain-free (BioRad) version contains a trihalo compound for fluorescent detection after UV irradiation with an imager (Gel Doc EZ System, BioRad).

### Construct design

The coding sequence for full-length human PCFT (gene: SLC46A1, UniProtKB entry: Q96NT5) followed by a C-terminal tandem 6X-histidine (His_6_) and a V5-epitope tag (HHHHHHGKPIPNPLLGLDST) [26] was subcloned into the baculovirus transfer vector pFastBac1™ (Thermo Fisher Scientific, Waltham, MA). The correct sequence and orientation of the insert was confirmed by sequencing (Genewiz, South Plainfield, NJ).

### Recombinant baculovirus generation

The PCFT recombinant baculovirus was generated using the Bac-to-Bac baculovirus expression system (Thermo Fisher Scientific) and was produced in Sf9 insect cells (Thermo Fisher Scientific) grown at 27°C in Grace’s medium (Thermo Fisher Scientific). The recombinant bacmid generation, transfection of insect cells, and amplification of baculovirus stock were performed following the manufacturer’s instructions (Thermo Fisher Scientific: Publication Number MAN0000414). The titer of the P3 virus stock was determined using BacPaK Baculovirus Rapid Titer Kit (Clontech) and the virus was stored at 4°C.

### PCFT expression

For optimization of PCFT expression, Sf9 cells in suspension were grown at 27°C in 250-ml baffled flasks containing 50 ml of HyClone CCM3 medium (GE Healthcare Life Sciences, Pittsburgh, PA), shaken at 125 rpm. The cells were infected at a density of 2 x 10^6^ cells/ml using a multiplicity of infection (MOI) of 2. One-ml samples were collected at indicated times to measure viability (Trypan blue staining), and PCFT expression was evaluated by Western blot using an antibody against the His_6_ tag at the C-terminus of the recombinant PCFT. After incubation with goat anti-mouse secondary antibody, Alexa Fluor 680 (Thermo Fisher Scientific), the signal was visualized using an imager (Odyssey Infrared Imager Li-Cor Biosciences, Lincoln, NE; or Typhoon FLA 9000 Biomolecular Imager, GE Healthcare Life Sciences). For PCFT expression, Sf9 cells were grown at 27°C in 2-l baffled flasks containing 750 ml of HyClone CCM3 medium. Cells were harvested by centrifugation (1,000 g for 15 min) ~48 h post-infection, when cell viability determined by Trypan blue staining was 40%.

### Sf9 membrane vesicle preparation

Membrane vesicles were prepared from Sf9 cells infected with recombinant baculovirus as described previously [28], with all steps performed at 4°C unless specified otherwise. The cells were resuspended in lysis buffer (150 mM NaCl, 50 mM Tris/HCl, pH 7.4, with 10 μM chymostatin, 10 μM leupeptin, 1 μM pepstatin A and 0.2 mM phenylmethylsulfonyl fluoride (PMSF)) and disrupted with a homogenizer (EmulsiFlex-C3, Avestin, ON, Canada; 4,000 psi, 4°C). The homogenate was clarified by centrifugation at 6,500 g for 15 min, followed by high-speed centrifugation at 125,000 g for 1 h to collect the crude membranes. The crude membranes were resuspended in lysis buffer using a Dounce homogenizer and the amount of total protein was quantified with the BCA Protein Assay Kit (Thermo Fisher Scientific) or Quick Start Bradford Assay Kit (BioRad).

### Deglycosylation with PNGase

PCFT was deglycosylated in non-denaturating conditions using recombinant Peptide-*N*-Glycosidase F, PNGase F (New England Biolabs, Ipswich, MA) following the manufacturer’s protocol. PNGase F (2,500 U) and GlycoBuffer 2 10X were mixed with crude membranes containing PCFT (150 μl at 3 mg/ml). The sample was incubated at 37°C overnight with gentle mixing, run on a 4–20% SDS-PAGE gel, and analyzed by Western blot as described earlier.

### Solubilization screening

The detergent n-dodecyl-β-D-maltoside (DDM) was purchased from Inalco Pharmaceuticals (San Luis Obispo, CA), whereas all other detergents used were from Anatrace (Maumee, OH): n-undecyl-β-D-maltopyranoside (UDM), n-decyl-β-D-maltopyranoside (DM), n-octyl-β-D-glucoside (OG), n-nonyl-β-D-glucoside (NG), 3[(3-cholamidopropyl) dimethylammonio] propanesulfonic acid (CHAPS), n-dodecyl-N,N-dimethyl-3-ammonio-1-propanesulfonate (AZ), 2,2-dioctylpropane-1,3-bis-β-D-maltopyranoside (DMNG), and n-dodecylphosphocholine (FS-12). For solubilization analysis, 10 μL of detergent mixture (2X of desired concentration (w/v)) dissolved in 500 mM NaCl, 50 mM Tris/HCl, pH 8, with 10% glycerol, was combined with 10 μl of membrane vesicles (4 mg/ml) diluted in the same buffer. The mixture was incubated at 4°C for 2 h with gentle rotation, followed by centrifugation at 125,000 g for 45 min to separate solubilized from unsolubilized material. Samples were subjected to SDS-PAGE and Western blot analysis as described earlier. ImageJ software was used for quantitative analysis.

### PCFT purification

Crude membranes at 1–2 mg/ml were solubilized for 2 h with 1% DDM (w/v) at 4°C, with gentle rotation, in 500 mM NaCl, 50 mM Tris/HCl, pH 8, with 10% glycerol, 10 μM chymostatin, 10 μM leupeptin, 1 μM pepstatin A, 0.2 mM PMSF, 0.1 mM tris-(2-carboxyethyl)phosphine (TCEP), and 1 mM 2-mercaptoethanol (2-ME). Unsolubilized material was removed by centrifugation at 125,000 g for 30 min, and the solubilized supernatant was incubated with TALON Co^2+^ affinity resin (Talon Superflow, Clontech) overnight, with gentle rotation, at 4°C. The resin was washed with 13 column volumes of 150 mM NaCl, 50 mM Tris/HCl, pH 8, with 10% glycerol, 0.1% DDM (w/v), 0.1 mM TCEP, 1 mM 2-ME and 10 mM imidazole, and was eluted with 200 mM imidazole in the same buffer. Peak fractions were collected and concentrated 2-fold using 30 kDa molecular mass cut-off centrifugal filters (Amicon Ultra, Millipore, MA).

### PCFT Size-Exclusion Chromatography (SEC) analysis

PCFT purified as described above was diluted 1/5 with SEC buffer (150 mM NaCl, 50 mM Tris/HCl, pH 7.4, with 10% glycerol, 0.02% Na azide, 0.1 mM TCEP and 1 mM 2-ME), concentrated 2- to 5-fold to ~0.9 mg/ml through 30 kDa molecular mass cut-off centrifugal filters (Amicon Ultra), filtered through a 0.22-μm syringe filter, and 500 μl were injected without delay into a Superdex 200 10/300 GL column (GE Healthcare Life Sciences) equilibrated with SEC buffer containing 0.1% DDM (w/v), at a flow rate of 0.5 ml/min. Proteins were detected by absorbance at 280 nm (*A*_280_). For calibration standard proteins were used as per manufacturer’s recommendations (GE Healthcare Life Sciences). Blue dextran 2000 was used to determine the void volume (V_0_) of the column. The value of the gel-phase partition coefficient (***K***_**av**_) was calculated for each standard as well as PCFT using the equation:

$$K_{av}=\frac{V_{e}-V_{0}}{V_{c}-V_{0}}$$

where ***V***_**e**_ is the elution volume, ***V***_**0**_ is the column void volume (8.16 mL), and ***V***_**c**_ is the geometric column volume (24 mL). For standard proteins, the calculated ***K***_**av**_ values were plotted against the log of their respective known molecular weight (Log *M*_r_). The linear equation

$$y=y_{0}+a\times x$$

was then used to predict the molecular weight (***x***) of PCFT, where ***a*** is the slope and ***y***_**0**_ is the y-intercept.

### Lipid reconstitution

Phosphatidylcholine (PC), phosphatidic acid (PA) and cholesterol (CH) (Avanti Polar Lipids, Alabaster, AL) were dissolved in chloroform under argon, mixed in 3:1:1 proportion, respectively (w/w), and the chloroform was evaporated. The dried lipids were resuspended at 1 mg/ml in 150 mM NaCl, 50 mM Tris/HCl, pH 7.4, with 0.1% DDM (w/v). PCFT was mixed with the lipids mixture at a 1:100 molar ratio, and proteoliposomes were formed by removing the detergent by overnight incubation with at 200 mg/ml Bio-Beads SM Adsorbents (BioRad). Liposomes and PCFT-proteoliposomes were then extruded through 0.2 μm polycarbonate membrane filters (Whatman Nuclepore Hydrophilic Membrane, track-etched, GE Healthcare Life Sciences) using a mini extruder (Avanti Mini Extruder, Avanti Polar Lipids) to produce unilamellar liposomes and proteoliposomes for uptake studies. The protein concentration in the proteoliposomes was 0.46 mg/ml.

## Functional assays

### Whole cell assay

Uptake was performed following previously published protocols for PCFT [29] and adherent Sf9 cells [30]. Sf9 cells were seeded as adherent cultures on 12-well plates (2 x 10^6^ cells/well; Falcon 353503, 12 Well TC-Treated Polystyrene, Corning Inc., Corning, NY). Seventy h after infection the cells were washed twice with HEPES-buffered saline (HBS: 150 mM NaCl, 2.5 mM MgCl_2_, 25 mM HEPES/NaOH, pH 7.4). Uptake was determined in 2 mL of MES buffered saline (MBS: 150 mM NaCl, 2.5 mM MgCl_2_, 25 mM MES/NaOH, pH 5.5) containing 1.94 μCi ^3^H-folic acid at 500 nM final concentration (specific activity 19.4 Ci/mmol, Moravek). Uptake was stopped after 10-min incubation by removing the MBS with ^3^H-folic acid and washing 4–5 times with 2 ml of ice-cold HBS. Scintillation fluid (Bio-Safe II Complete Counting Cocktail, RPI, Mount Prospect, IL) was added to the samples after cell lysis with 0.1 M NaOH, and the radioactivity was counted on a scintillation counter (PerkinElmer Tri-carb liquid scintillation analyzer, Waltham, MA). For concentration-dependent uptake studies we used 0.038 μCi ^3^H-folic acid at 1 nM final concentration and 1.94 μCi ^3^H-folic acid at 100 nM to 10 μM final concentrations.

### Uptake in liposomes and proteoliposomes

Liposomes or proteoliposomes (5 μl) were diluted in 95 μl of 140 mM NaCl, 2.8 mM KCl, 1 mM CaCl_2_, 2 mM MgCl_2_, 10 mM MES/OH, pH 5.5, containing 0.058 μCi ^3^H-folic acid at 300 nM final concentration (specific activity 19.4 Ci/mmol, Moravek). After 30 s, 2 ml of ice-cold stop solution was added (140 mM NaCl, 2.8 mM KCl, 2 mM MgCl_2_, 1 mM CaCl_2_, 10 mM HEPES/NaOH, pH 7.4), and the mixture was applied under vacuum to 0.05-μm membrane filters (22 mm diameter, VMWP02500, Millipore MF, Billerica, MA) held in standard glass filter assemblies with fritted base, and washed 3–4 times with 2.5 ml ice-cold stop solution. Membrane filters were soaked and vortexed in scintillation fluid, and the radioactivity was counted.

## Results and discussion

### Optimization of PCFT expression

**Fig 1:**
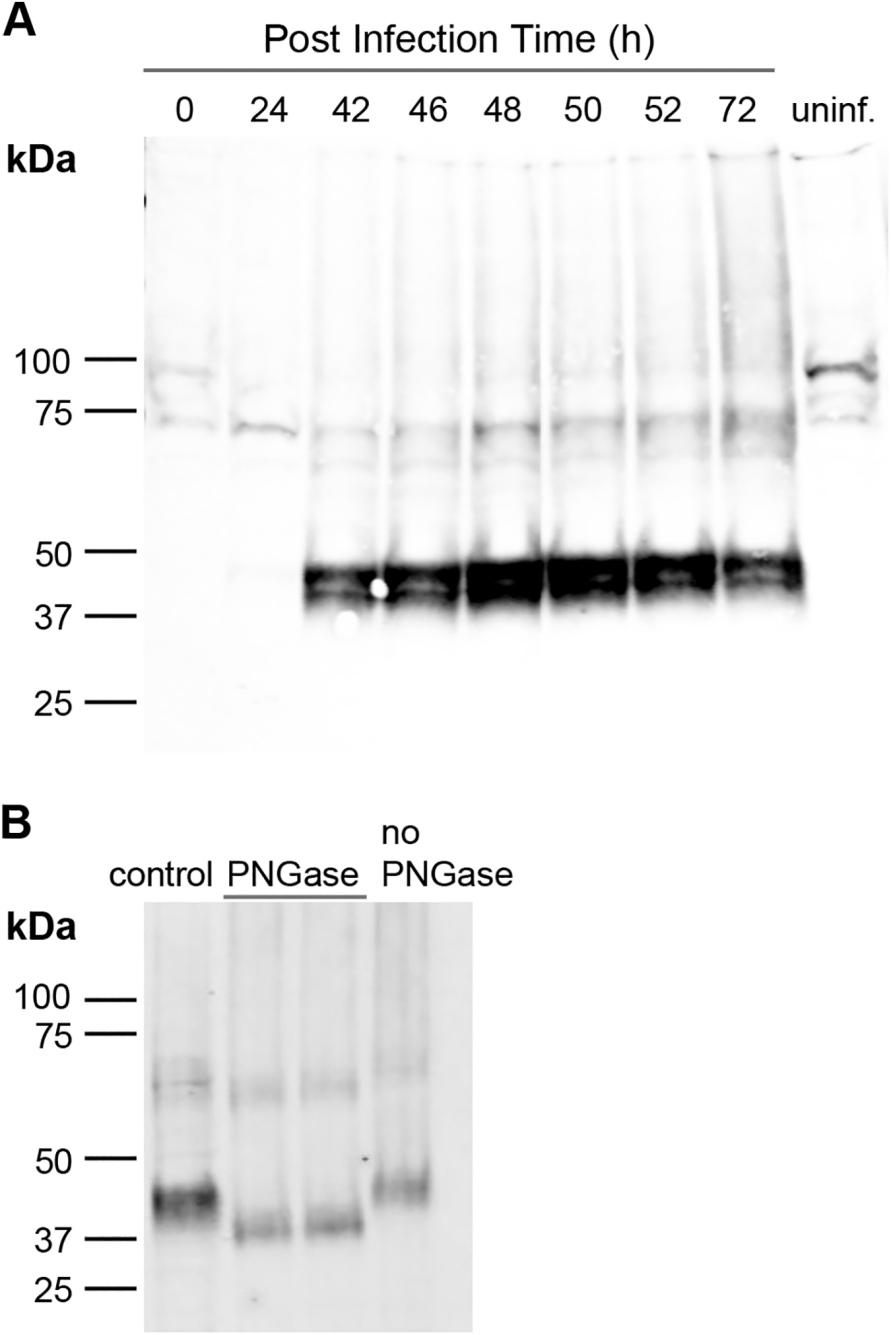
PCFT expression in Sf9 cells. A. Sf9 cells in 50 ml suspension culture at a density of 2 x 10^6^ cells/ml were infected at a MOI of 2. One-ml whole cell samples were collected at the indicated time points, electrophoresed on a 4–15% Mini Protean TGX Precast SDS-PAGE gel (BioRad), transferred to a PVDF membrane, and immunoblotted using an antibody against the His_6_ tag of PCFT. PCFT bands were observed at ~39 and ~43 kDa. The highest PCFT expression was observed 48 h post infection. No PCFT expression was observed at the time of infection (0 h) or in uninfected cells after 48 h (uninf). B. Treatment of membrane vesicles with PNGase F under non-denaturing conditions shifted the PCFT band from ~43 kDa to ~39 kDa (each lane treated with PNGase corresponds to a different sample preparation).

In pilot studies where we varied the number of viral particles per cell (multiplicity of infection, MOI) and density of Sf9 cells grown in a 50-ml suspension culture, we found that PCFT expression was better at a MOI of 2 and a density of 2 x 10^6^ cell/ml. Fig 1A shows PCFT expression under these conditions, as a function of time after infection. For each timed sample, we determined cell viability and PCFT expression by Western blot. PCFT expression peaked 48 h post-infection with a cell viability of ~40%. In summary, the following conditions yielded the best PCFT expression: Sf9 cells infected at a density of 2 x 10^6^ cell/ml with a MOI of 2 and harvested 48 h after infection.

The whole cell samples used for the initial expression investigations yielded two close bands (~39 and ~43 kDa) in Western blots. N-linked glycosylation of two asparagine residues within the first extracellular loop has been observed in recombinant human PCFT expressed in mammalian cells or *Xenopus laevis* oocytes [26, 31]. In these expression systems, treatment with PNGase F shifted the PCFT band in Western blots from ~55 to ~35 kDa. Insect cells can add compact, relatively homogenous α1–6 fucosylated Man_3_GlcNAc_2_ sugar moieties [32] of ~16 kDa per glycosylation site [33]. Therefore, we suspected that the band with slower mobility in our blots corresponds to glycosylated PCFT localized at the plasma membrane, whereas the faster band corresponds to non-glycosylated PCFT. Isolation of membrane vesicles and solubilization yielded samples highly-enriched in the 43 kDa species (Fig 1B; control). Consistent with glycosylation, PNGase treatment shifted the slower mobility band to a band of higher mobility (Fig 1B). In our hands, PCFT bands obtained from Sf9 whole cell lysates correspond to approximate molecular weights of 43 for glycosylated PCFT and 39 for deglycosylated PCFT.

### Detergent screening for solubilization of PCFT

**Fig 2:**
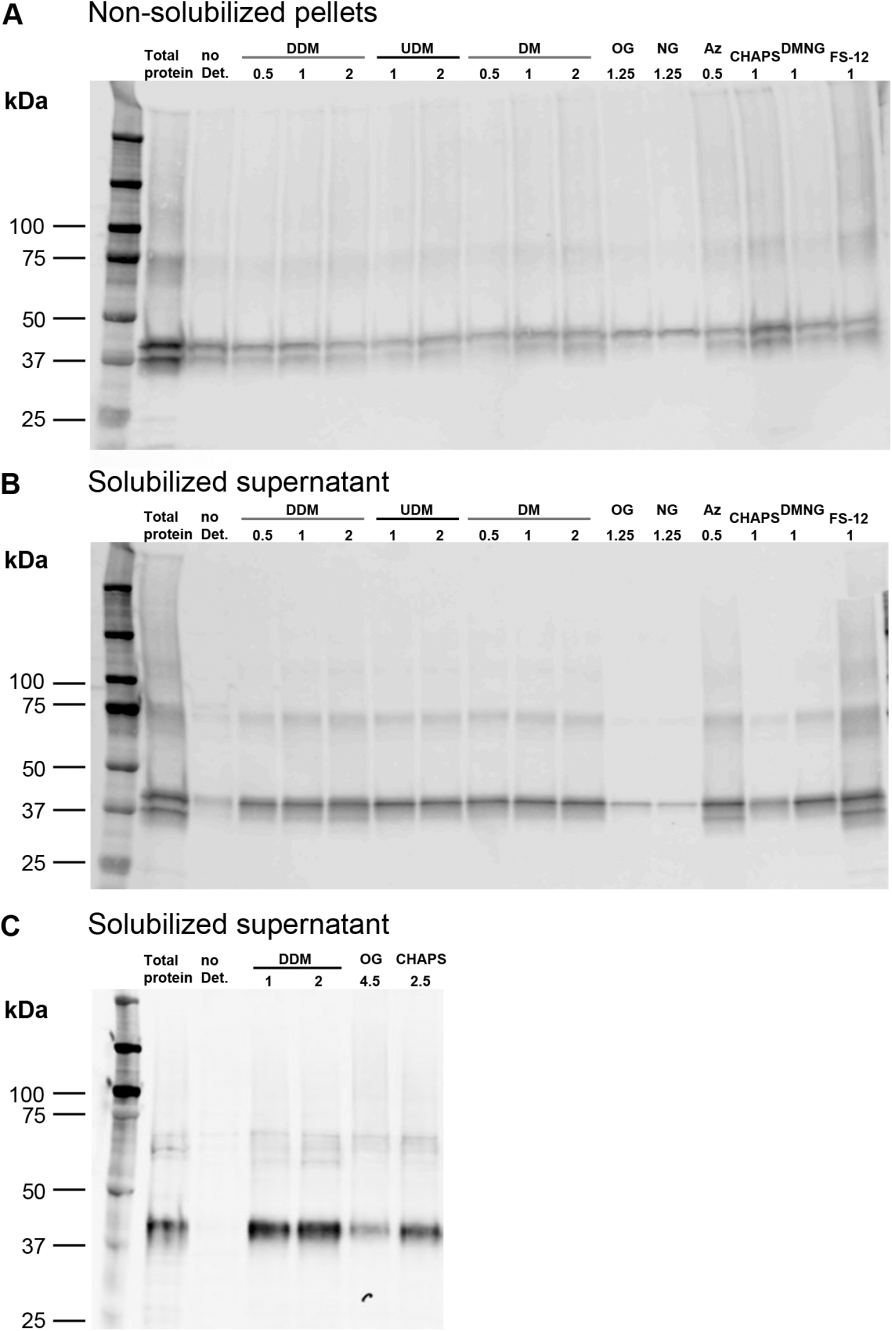
Detergent screening for solubilization of PCFT. Nine different detergents were used at the indicated concentrations to analyze solubilization of PCFT from membranes isolated from Sf9 cells. After a 2-h incubation, solubilized supernatants and pellets were analyzed using 4–15% MiniProtean TGX Precast SDS-PAGE gels, transferred to PVDF membranes and immunoblotted for detection with an antibody against the His_6_ tag of PCFT. First lane (Total protein) is the total amount of PCFT in Sf9 crude membranes before solubilization, and the second lane is a sample without detergent. (A) Non-solubilized pellets of the initial screen, (B) solubilized supernatants of the initial screen, and (C) solubilized supernatants with increased OG and CHAPS concentrations. See text for detergent abbreviations.

DDM has been widely used as a detergent to solubilize and study membrane proteins, including MFS transporters [34–43]. In pilot experiments to identify detergents suitable for solubilization of PCFT from Sf9 membranes we tested nine different detergents (concentrations in %; w/v), including nonionic (0.5–2% DDM, 1–2% UDM, 0.5–2% DM, 1.25% and 4.5% OG, 1.25% NG, 1% DMNG) and zwitterionic detergents (0.5% AZ, 1% and 2.5% CHAPS and 1% FS-12). PCFT in Sf9 membranes was solubilized with these detergents, centrifuged at high-speed to separate solubilized proteins (supernatant) from unsolubilized material (pellet), separated by SDS-PAGE, and visualized by Western blot. The efficiency of solubilization was assessed by comparing the intensity of the PCFT band after solubilization to that of the control band (unsolubilized, non-centrifuged sample). PCFT in the Sf9 membranes was solubilized quantitatively (~90 to 100%) with 1 or 2% DDM, 1% FS-12 or 0.5% AZ (Fig 2). DDM consistently yielded very high efficiency in solubilization (> 70%). Based on this and its widespread use, we used DDM for all subsequent preparations.

### Affinity purification

**Fig 3:**
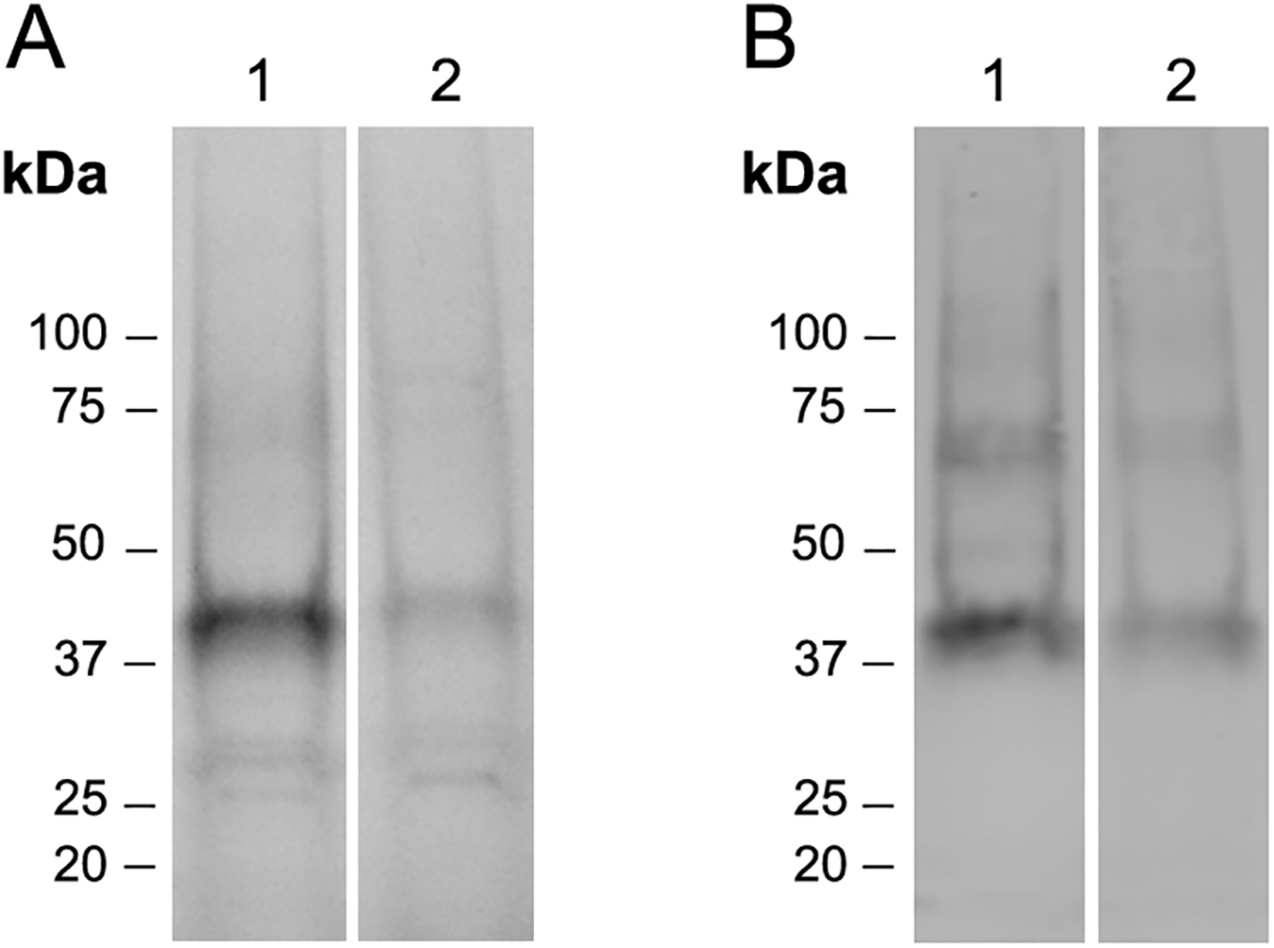
Lipid reconstitution of PCFT. PCFT was reconstituted in liposomes as described in Experimental Procedures. Purified protein and reconstituted PCFT were subjected to SDS-PAGE (4–15% MiniProtean TGX Precast gel). (A) Protein staining (Stain-free gel, BioRad) and (B) Western blot of the same gel analyzed using an antibody against the His_6_ tag of PCFT. Lane 1: purified protein eluted from the Talon Co^2+^ resin; lane 2: PCFT reconstituted in proteoliposomes.

We enriched PCFT from solubilized membrane proteins by liquid chromatography based on the affinity of its His_6_ tag for transition metals. The PCFT eluted from a TALON Co^2+^ resin was significantly cleaner than that eluted from a resin containing immobilized Ni^2+^ (data not shown). The His_6_-tagged PCFT eluted from the Co^2+^ resin with 200 mM imidazole migrated at ca. 43 kDa in a 4–15% MiniProtean TGX Precast SDS-PAGE gel and showed a high degree of purity (Fig 3).

### Size-Exclusion Chromatography (SEC) analysis

**Fig 4:**
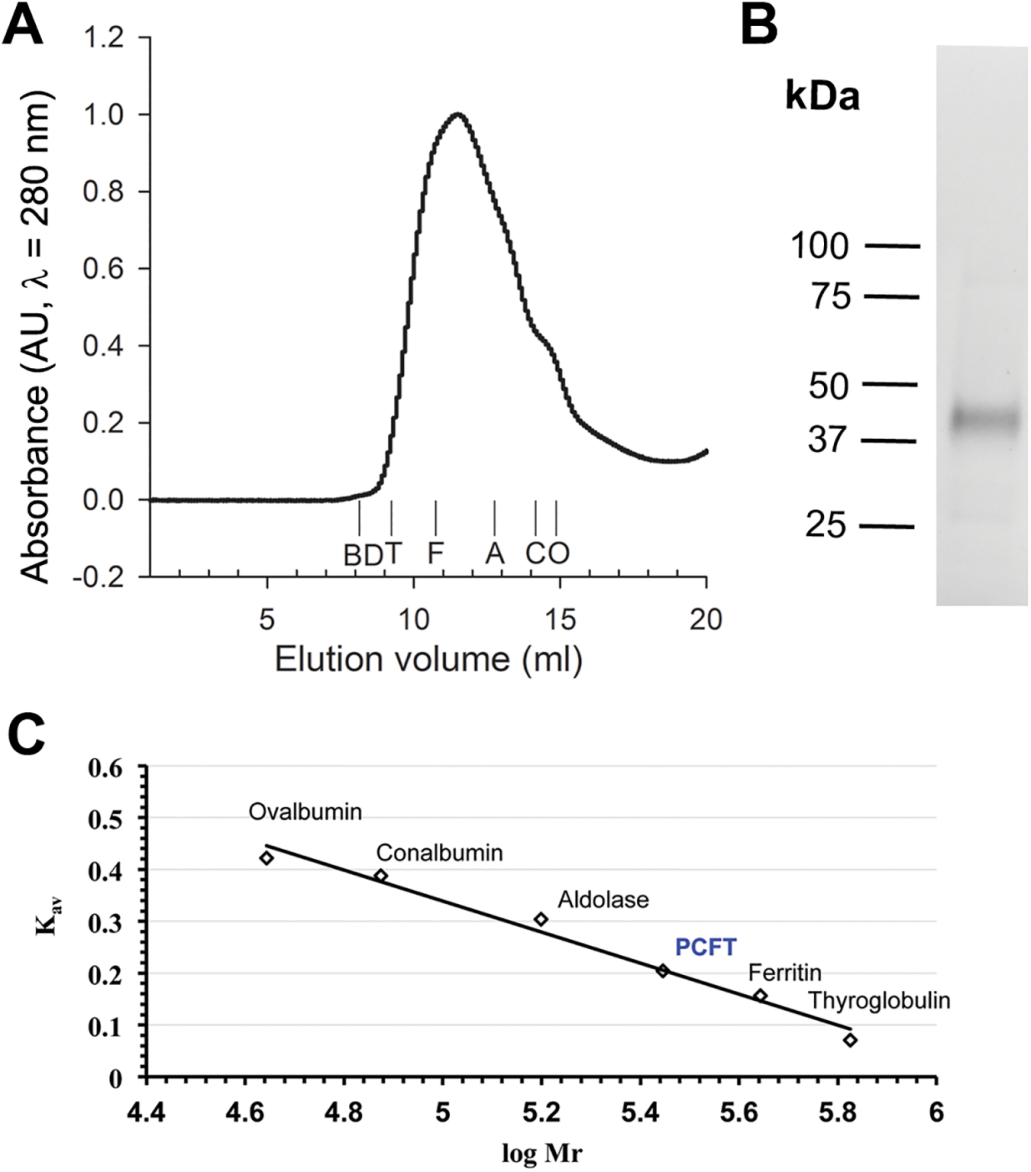
Size-exclusion chromatography analysis of PCFT. (A) Elution profile of PCFT solubilized in DDM. Elution volumes of standard proteins are indicated as follows: thyroglobulin (T, 669 kDa), ferritin (F, 440 kDa), aldolase (A, 158 kDa), conalbumin (C, 75 kDa), ovalbumin (O, 44 kDa). Blue dextran (BD, 2 MDa) was used for void volume determination. (B) PCFT fraction corresponding to the peak PCFT elution fraction was analyzed by protein staining (BioRad stain-free imaging). (C) The partition coefficients (K_av_) of the standard proteins are plotted against the log of their molecular weights to calculate the size of the PCFT-DDM complex, yielding an apparent size of 280 kDa.

DDM-solubilized PCFT purified by immobilized Co^2+^ affinity chromatography was purified further by SEC, with an overall yield of pure PCFT of ~0.9 mg/l culture. Based on PCFT’s elution volume of 11.4 ml and the elution profiles of protein standards, the calculated molecular mass of the PCFT-DDM complex was ~280 kDa (Fig 4) consistent with a large amount of detergent, as has been observed previously for other 12 transmembrane segment transporters [44]. Alternatively, our purified PCFT could be an oligomer, but this seems unlikely because we have shown that the monomer is the human PCFT structural/functional unit in membranes of mammalian cells and frog oocytes [26].

### Functional characterization

#### Specific uptake of folic acid in Sf9 cells

**Fig 5:**
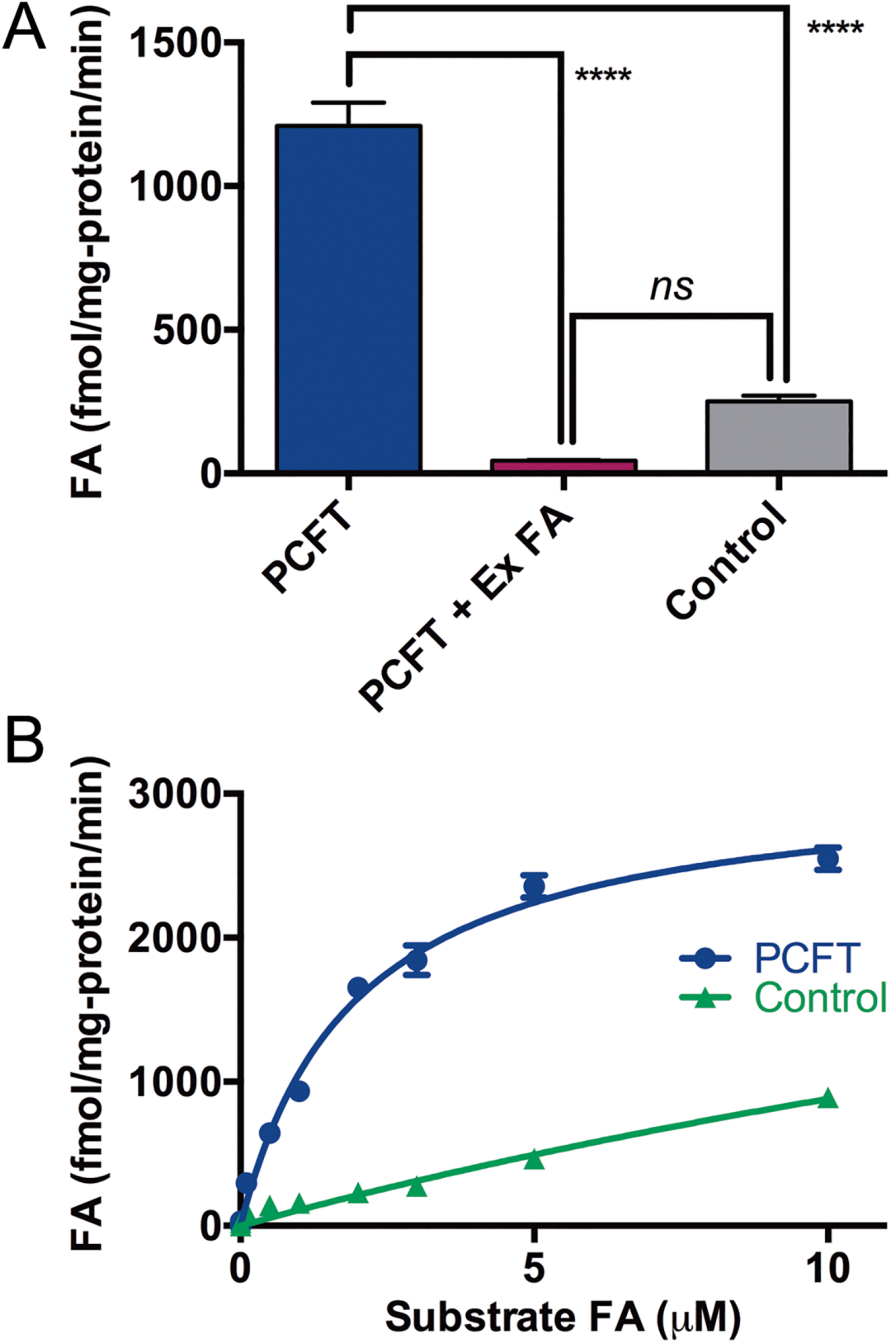
PCFT-dependent ^3^H-folic acid uptake in Sf9 cells. (A) 10-min uptake of 500 nM ^3^H-folic acid (FA) by PCFT-expressing Sf9 cells determined at pH 5.5. Data are means ± SD. The value in Sf9 cells expressing PCFT (PCFT) was significantly different from that of uninfected cells (Control) (1-way ANOVA with Tukey’s multiple comparison test, P ≤ 0.0001, ****). Uptake was reduced significantly in the presence of a 200-fold excess of unlabeled folic acid (PCFT + Ex FA) (1-way ANOVA with Tukey’s multiple comparison test, P ≤ 0.0001, ****). The difference between the PCFT + Ex FA and Control was not significant (ns). (B) Concentration dependence of the ^3^H-folic acid uptake in PCFT-expressing Sf9 cells (PCFT) and uninfected cells (Control). Data was fit using the Michaelis Menten equation (Graphpad Prism, San Diego, CA).

The uptake of ^3^H-folic acid in Sf9 cells expressing PCFT and uninfected cells was measured over 10 min at pH 5.5 [29]. The time course of folic acid uptake was not examined in the present study, but Fig 5A shows that the uptake in cells expressing PCFT was significantly higher than in uninfected cells, and was reduced in the presence of a 200-fold excess of cold (unlabeled) folic acid (one-way ANOVA with Dunnett’s multiple comparison test, P < 0.0001). These data indicate that PCFT expressed at the plasma membrane in Sf9 cells is functional. Fig 5B shows that the uptake measured at pH 5.5 over 10 min was concentration dependent, with a K_m_ for ^3^H-folic acid uptake of 1.94 ± 0.20 **μ**M (n = 3). This K_m_ is similar to that reported in mammalian cells (1.7 **μ**M in HEK 293 cells)[29] and *X*. *laevis* oocytes (1.3 **μ**M)[9].

#### Functionality of lipid reconstituted PCFT

**Fig 6:**
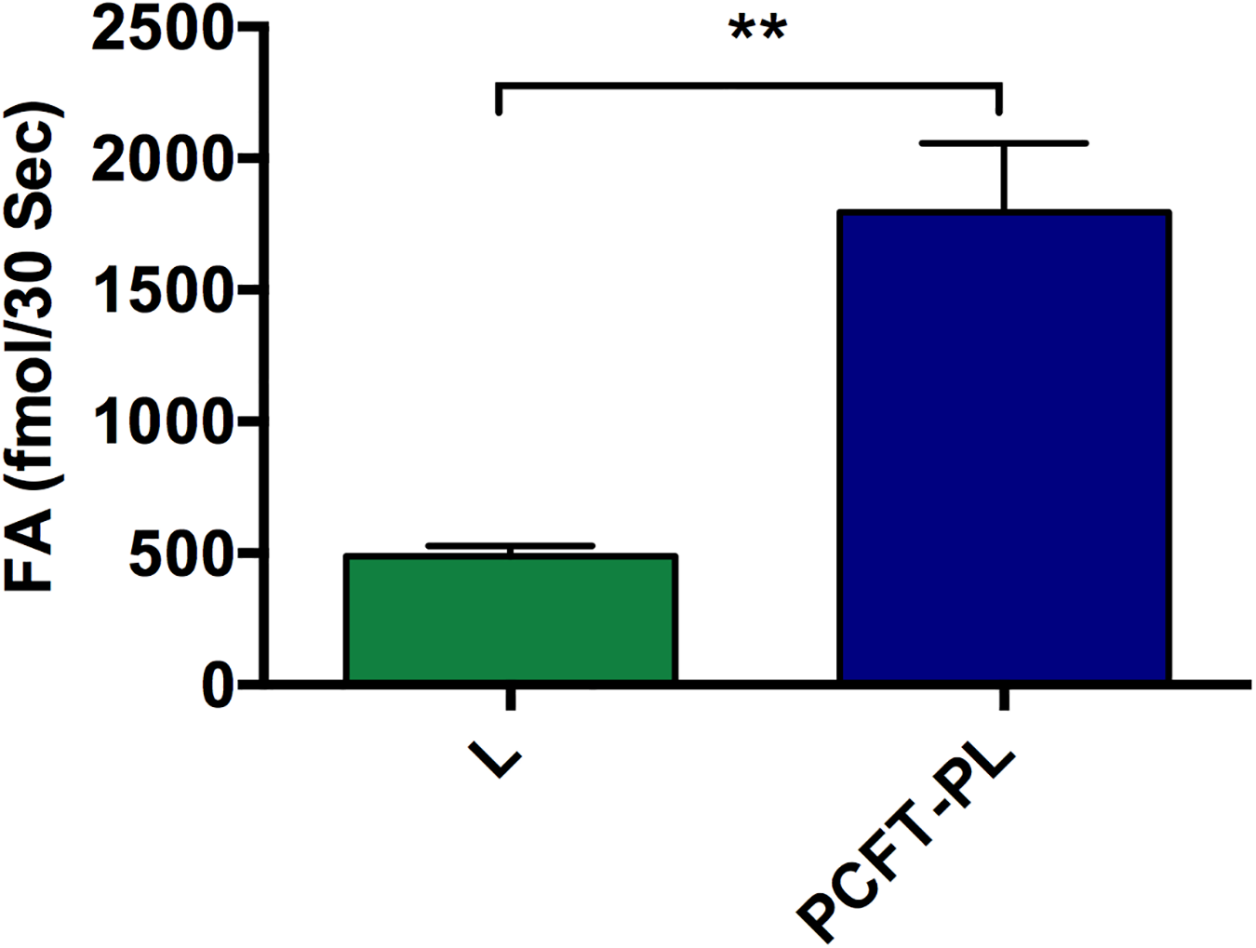
^3^H-folic acid uptake by purified PCFT reconstituted in liposomes. The 30-s uptake of 300 nM ^3^H-folic acid (FA) into unilamellar PCFT-proteliposomes (PCFT-PL) was measured at pH 5.5. Unilamellar empty liposomes (L) served as control. The uptake in PCFT-PL was significantly higher than that in liposomes (t-test: L–PCFT-PL P = 0.008, r^2^ = 0.86; one preparation, n = 3).

Affinity purified PCFT was concentrated to 0.6 mg/ml and reconstituted in unilamellar liposomes as indicated under Experimental Procedures. The protein staining of SDS-PAGE gel and Western blot analysis in Fig 3 show the presence of PCFT in the proteoliposomes. PCFT function was demonstrated by the 30-s uptake of 300 nM ^3^H-folic acid (Fig 6), where PCFT-proteoliposomes showed significantly higher uptake of ^3^H-folic acid than liposomes (t-test: L vs. PCFT-PL P = 0.008, r^2^ = 0.86; one preparation, n = 3). These results demonstrate reconstitution of functional PCFT in liposomes.

## Conclusion

Here, we have shown expression of recombinant human PCFT in Sf9 cell membranes, and established procedures that yield highly-purified PCFT in sufficient amounts for functional, biochemical, biophysical and structural studies. We demonstrate that PCFT is functional in Sf9 cells and importantly remains functional after purification and reconstitution in liposomes. Sf9 cells have been the source for heterologous expression of a number of membrane proteins that yielded more than 100 high-resolution structures, including the eukaryotic SWEET transporter [45], the human glucose transporter GLUT1 [46], the ATP-gated P2X4 ion channel [47], the human P2Y1 receptor [48], the Cystic Fibrosis Transmembrane Conductance Regulator [49], several pentameric ligand-gated ion channels [28, 50], and G-protein coupled receptors [51]. Expression and purification of PCFT in Sf9 cells in a functional state is a key step towards providing PCFT not only for detailed structural studies, including crystallization trials, but also for mechanistic investigations. Ultimately, elucidating the molecular mechanism of transport of folate and antifolates through PCFT will require high-resolution structural information of PCFT in diverse functional states in combination with detailed biochemical and biophysical studies. The PCFT expression and purification in Sf9 cells reported here is therefore a valuable tool that opens up doors to the use of established structural, biophysical and biochemical techniques in the field of PCFT research.

## Abbreviations

AZ: n-dodecyl-N,N-dimethyl-3-ammonio-1-propanesulfonate
CHAPS: 3[(3-cholamidopropyl) dimethylammonio] propanesulfonic acid
Ch: Cholesterol
DDM: n-dodecyl-β-D-maltoside
DM: n-decyl-β-D-maltopyranoside
DMNG, 2: 2-dioctylpropane-1,3-bis-β-D-maltopyranoside
FS-12: n-dodecylphosphocholine
HBS: hepes buffered saline
His_6_: hexahistidine
2-ME: 2-mercaptoethanol
MBS: MES-buffered saline
MFS: major facilitator superfamily
MOI: multiplicity of infection
NG: n-nonyl-β-D-glucoside
OG: n-octyl-β-D-glucoside
PCFT: proton-coupled folate transporter
PC: Phosphatidyl choline
PA: Phosphatidic Acid
PMSF: phenylmethylsulfonyl fluoride
SEC: size exclusion chromatography
Sf9: *Spodoptera* *frugiperda*
SEC: size exclusion chromatography
TCEP: tris(2-carboxyethyl)phosphine
UDM: n-undecyl-β-D-maltopyranoside
